# Supplementary material for: MoChia1 is a GH18 reducing-end GlcNAc–releasing chitin oligosaccharide hydrolase from the rice blast fungus Magnaporthe oryzae
Source: J Biol Chem. 2026 Apr 17;302(6):111462. doi: 10.1016/j.jbc.2026.111462 (PMC13213659; doi:10.1016/j.jbc.2026.111462)
Supplement: Supporting information [file mmc1.docx]

**Supporting Information**

**MoChia1 is a GH18 Reducing-End GlcNAc–Releasing Chitin Oligosaccharide Hydrolase from the Rice Blast Fungus *Magnaporthe oryzae***

Takayuki Ohnuma^1,2^, Shun Imaoka^1^, Chikara Kataoka^1^, Teruki Yoshimoto^1^, Ryuta Okada^1^, Toru Takeda^1^, Tamo Fukamizo^1^, Shohei Sakuda^3^, Makoto Ogata^4^

^1^Department of Advanced Bioscience, Kindai University, 3327-204 Nakamachi, Nara 631-8505, Japan

^2^Agricultural Technology and Innovation Research Institute (ATIRI), Kindai University, 3327-204 Nakamachi, Nara 631-8505, Japan.

^3^Department of Biosciences, Teikyo University, 1-1 Toyosatodai, Utsunomiya 320-8551, Japan

^4^Faculty of Food and Agricultural Sciences, Fukushima University, 1 Kanayagawa, Fukushima, Fukushima 960-1296, Japan

Corresponding author: Takayuki Ohnuma (e-mail, ohnumat@nara.kindai.ac.jp; Tel, +81-742-43-7297) and Makoto Ogata (e-mail, ogata@agri.fukushima-u.ac.jp; Tel, +81-24-503-4982)

Running title: Reducing-End GlcNAc–Releasing Chitin Oligosaccharide Hydrolase

**
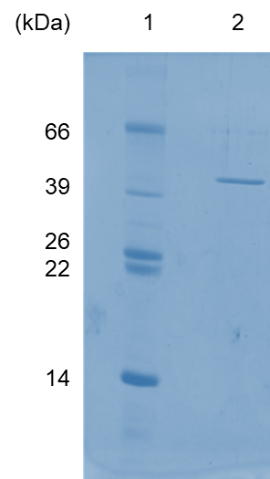
**

**Figure S1. SDS-PAGE of MoChia1.**

Lane 1: marker, Lane 2: purified MoChia1.


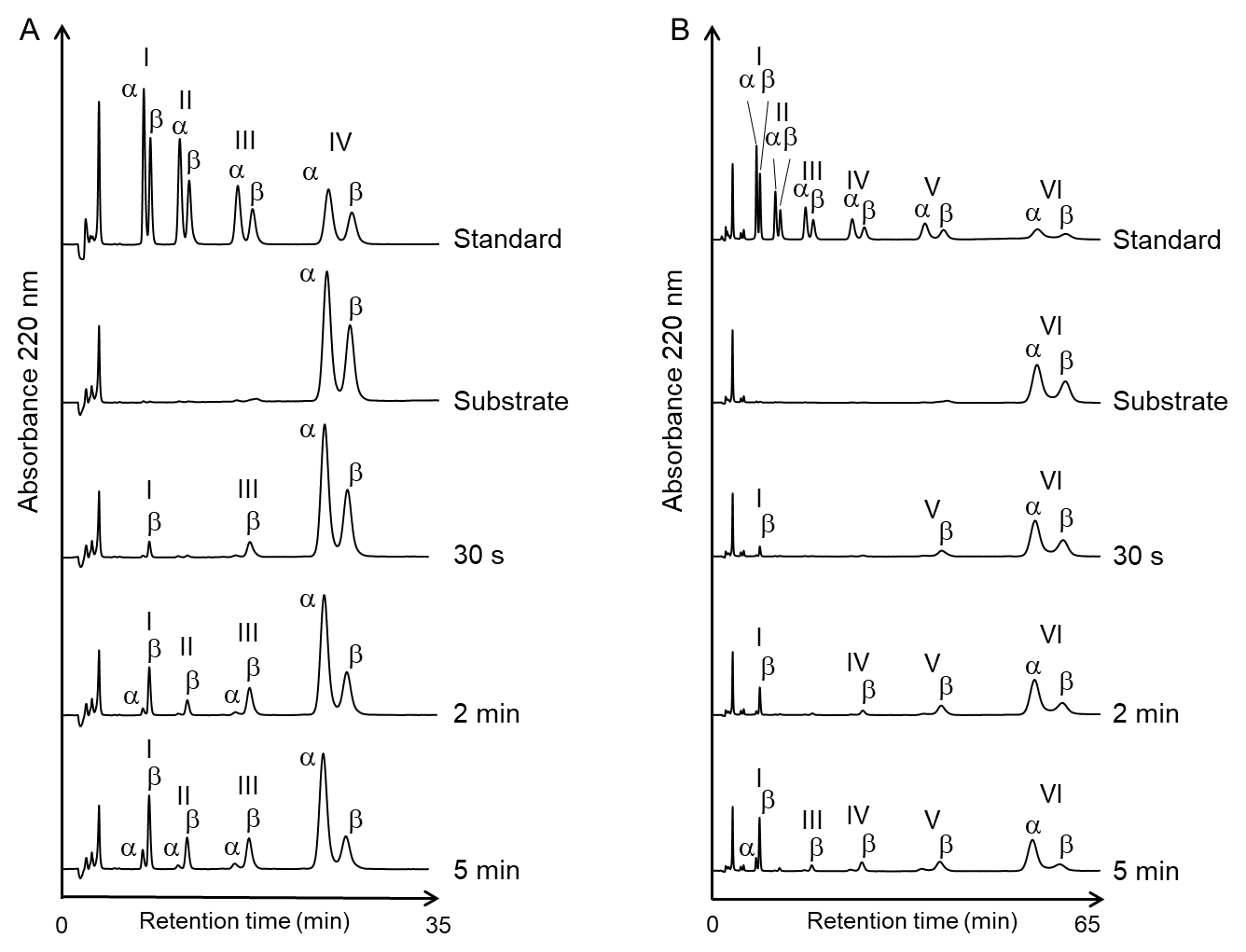


**Figure S2. Anomer formation catalyzed by MoChia1 from the (GlcNAc)_4_ and (GlcNAc)_6_ substrates.**

Time-dependent HPLC profiles showing the hydrolysis of (GlcNAc)_4_ (*A*) and (GlcNAc)_6_ (*B*) by MoChia1. Enzyme and substrate concentrations were 2.0 μM and 8 mM, respectively. The enzyme reaction was conducted in 20 mM sodium acetate buffer, pH 5.0, at 25°C. Numerals in the figure represent the degree of polymerization.


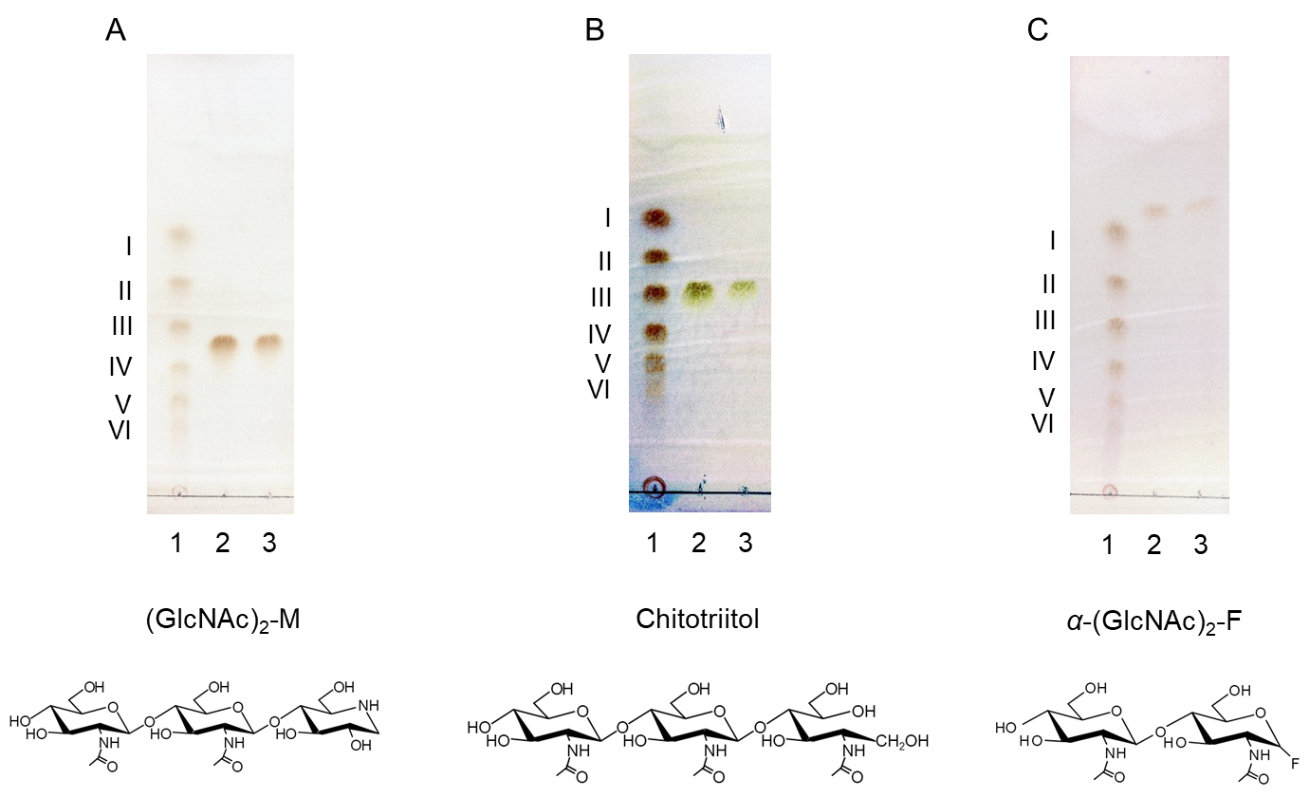


**Figure S3. TLC analysis of the hydrolysis of (GlcNAc)_2_-M, chitotriitol, and *α*-(GlcNAc)_2_-F by MoChia1.**

A reaction mixture comprising 2.0 μM MoChia1 and 8.0 mM of (GlcNAc)_2_-M (*A*), chitotriitol (*B*), and *α*-(GlcNAc)_2_-F (*C*) in 20 mM sodium acetate buffer, pH 5.0, was incubated for 1 h at 37°C. Reaction products were detected with methanol containing sulfuric acid. Lanes: 1, I–VI represent a standard mixture of (GlcNAc)*_n_* (*n* = 1–6); 2, substrate; 3, reaction product. The chemical structure of each substrate is shown below each TLC plate.


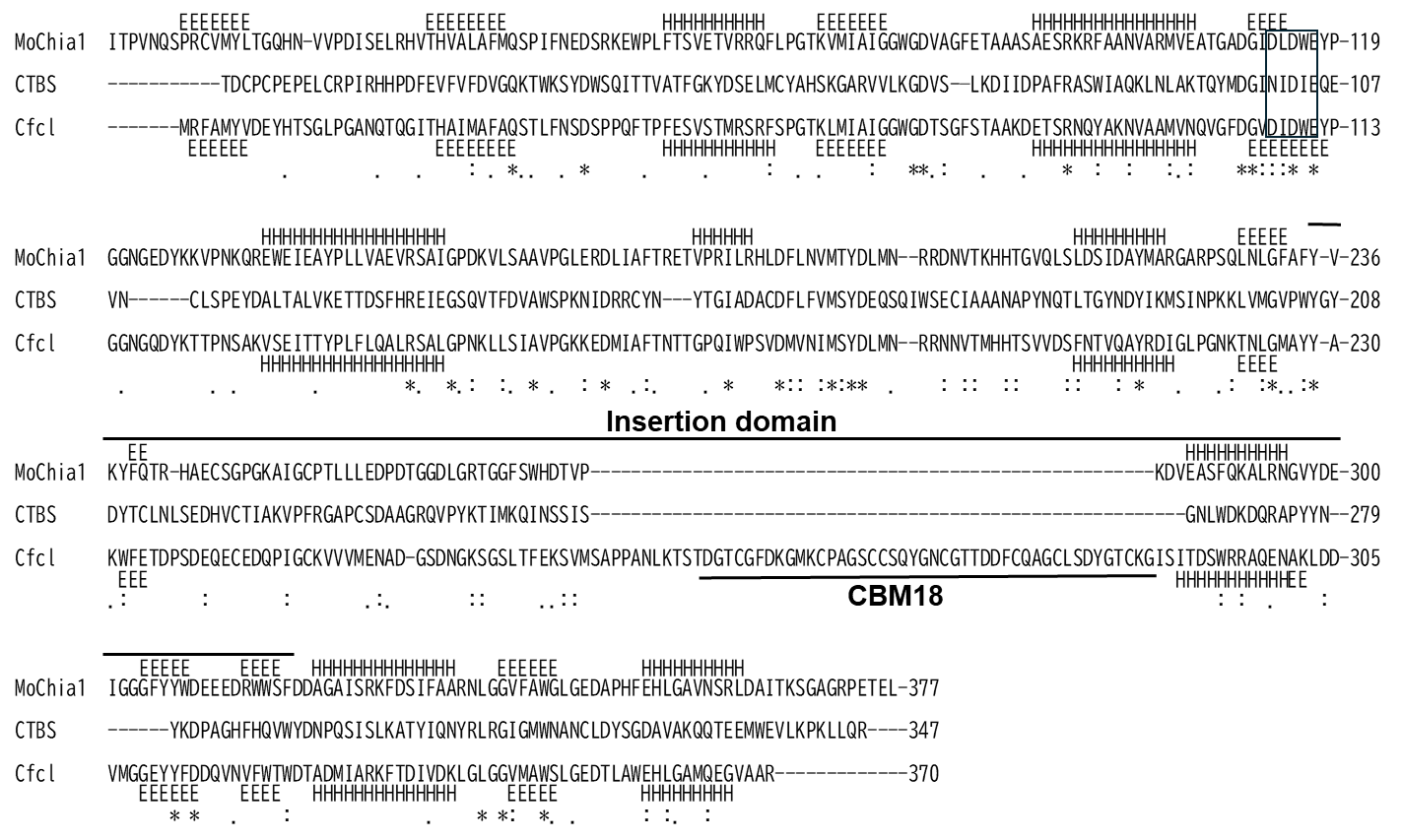


**Figure S4. Amino acid sequence alignment of GH18 chitinases that act on the glycosidic linkage at the reducing end of a chitin oligosaccharide.**

The alignment of MoChia1 (GenBank accession number: MGG_08054), CTBS from *Homo sapiens* (M95767.1) and Cfcl from *Aspergillus niger* (XM_001400452.2) was obtained using the ClustalW (47). Dashes indicate gaps. Identical residues are indicated by the symbol "*", two identical residues out of three amino acids are marked by the symbol ":", and similar amino acids are marked by the symbol ".". The box indicates the catalytic motif (D(N)xDxE), which is conserved in GH18 enzymes. The predicted secondary structures of MoChia1 and Cfcl, as determined by Jpred and AlphaFold (23,48), are shown above and below their respective sequences. H, *α*-helix; E, *β*-sheet. The positions of the putative MoChia1 (*α*+*β*) insertion domain and the Cfcl CBM18 domain are represented by solid lines above and below the sequences, respectively.

**
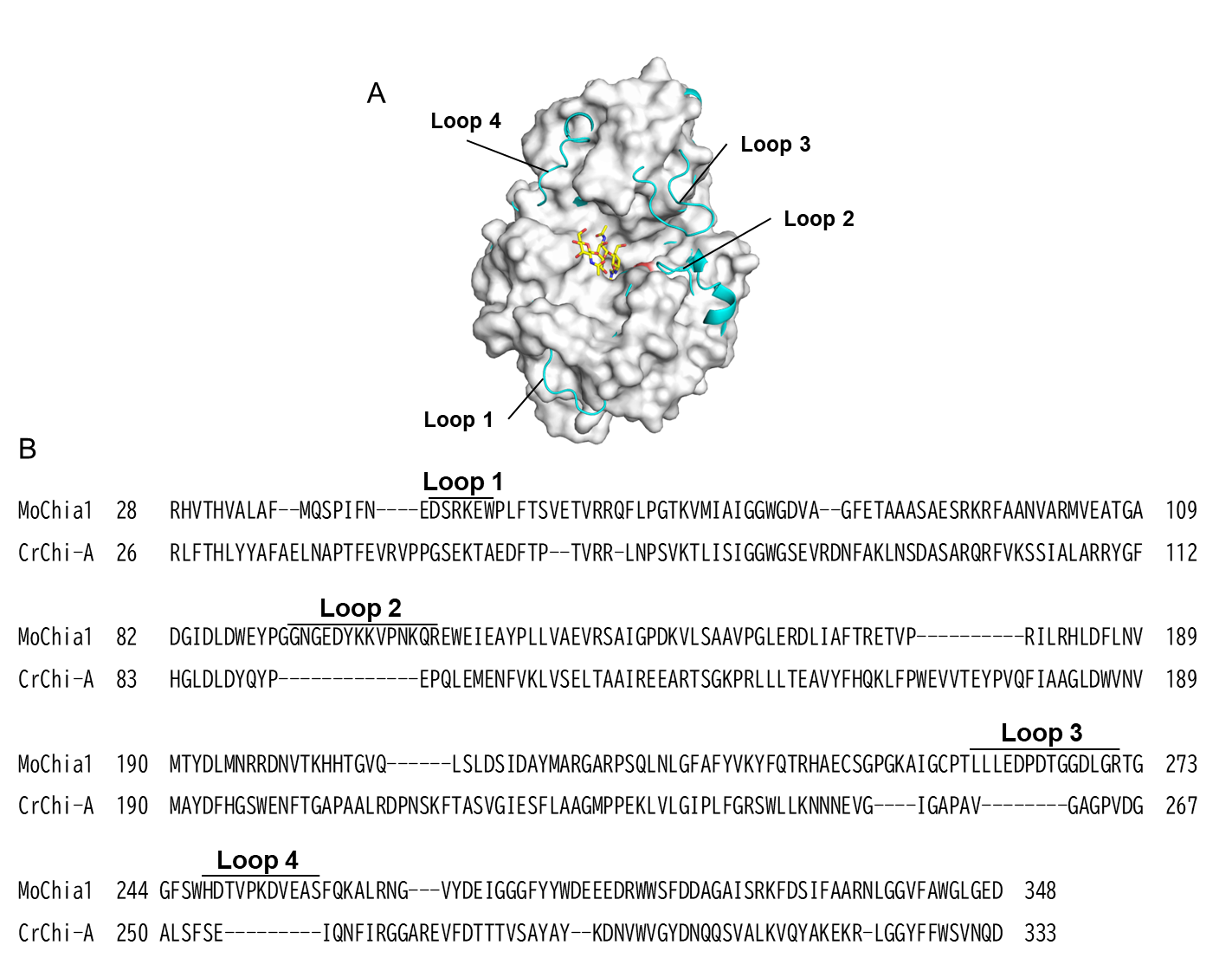
**

**Figure S5. A structural comparison of CrChi-A and MoChia1.**

(*A*), superimposed image of AlphaFold model of MoChia1 (cartoon, cyan) on CrChi-A–allosamidin complex. Extended loop structures (loop 1–4) found in MoChia1 were indicated. (*B*), alignment of the MoChia1 and CrChi-A sequences was obtained through PSI-BLAST analysis (49). The positions of the extended loops are indicated by solid lines above the sequence. The model of MoChia1was prepared by using AlphaFold (23). The three-dimensional structures were generated using PyMol (50).
